# Multi-laboratory comparison of three commercially available Zika IgM enzyme-linked immunosorbent assays

**DOI:** 10.1016/j.jviromet.2018.06.018

**Published:** 2018-06-30

**Authors:** Alison Jane Basile, Christin Goodman, Kalanthe Horiuchi, Angela Sloan, Barbara W. Johnson, Olga Kosoy, Janeen Laven, Amanda J. Panella, Isabel Sheets, Freddy Medina, Emelissa J. Mendoza, Monica Epperson, Panagiotis Maniatis, Vera Semenova, Evelene Steward-Clark, Emily Wong, Brad J. Biggerstaff, Robert Lanciotti, Michael Drebot, David Safronetz, Jarad Schiffer

**Affiliations:** a Division of Vector-Borne Diseases, Centers for Disease Control and Prevention, Fort Collins, CO, United States; b Zoonotic Diseases and Special Pathogens, National Microbiology Laboratory, Public Health Agency of Canada, Winnipeg, Manitoba, Canada; c Microbial Pathogenesis and Immune Response Laboratory, Division of Bacterial Diseases, Centers for Disease Control and Prevention, Atlanta, GA, United States; d Dengue Branch, Division of Vector-Borne Diseases, Centers for Disease Control and Prevention, San Juan, Puerto Rico, United States

**Keywords:** Zika, Dengue, Virus, Kits, ELISA, Enzyme-linked immunosorbent assay, Commercial, IgM, IgG, Immunoglobulin M, Immunoglobulin G, Comparison, Nonstructural protein 1, NS1, Envelope E

## 1. Introduction

Zika virus (ZIKV) is an enveloped, positive-sense RNA virus in the family *Flaviviridae*, genus *Flavivirus*. It was first discovered in rhesus monkeys in 1947 in the Zika Forest of Uganda (Dick et al., 1952) and historically of unclear importance given the rarity of reported cases and to relatively mild symptoms in humans. The virus is chiefly transmitted by *Aedes* mosquitoes, the carrier of other flaviviruses of medical importance such as the dengue viruses (DENVs) and yellow fever virus (YFV). Little research had been conducted on ZIKV prior to a 2007 outbreak in Yap, Federated States of Micronesia (Duffy et al., 2009), at which point the virus was sequenced and molecular and serological tests were developed (Lanciotti et al., 2008).

Zika virus was identified in French Polynesia in 2013 (Cao Lormeau et al., 2014), and Easter Island in 2014 (Tognarelli et al., 2016). Following this, an extensive epidemic began in late 2015 first identified in Natal, northeastern Brazil (Zanluca et al., 2015). The virus subsequently spread widely among Central/South America and the Caribbean (Wikan and Smith, 2016), with small clusters of local disease transmission identified in the United States in Florida and Texas (Khawar et al., 2017). Sequencing identified that the ZIKV currently circulating in the Americas is derived from the Southeast Asian genotype (Brasil et al., 2016). The discovery of increased incidence of microcephaly and other birth defects in newborns (Fitzgerald et al., 2018) took ZIKV from being considered as relatively benign to being a critical public health concern, causing the World Health Organization (WHO) to declare a public health emergency in 2016 (Gulland, 2016).

The CDC Zika IgM-antibody capture enzyme-linked immunosorbent assay (Centers for Disease Control and Prevention Zika MAC-ELISA) was developed for detection of ZIKV immunoglobulin M (IgM) at the CDC in 2007 (Lanciotti et al., 2008), and was recognized as highly cross-reactive with other members of the flavivirus genus. The CDC Zika MAC-ELISA, utilizing inactivated whole virus ZIKV antigen, was used for serological diagnosis during the Yap outbreak in combination with reverse-transcriptase polymerase chain reaction (RT-PCR) in acute samples. The CDC Zika MAC-ELISA was granted Emergency Use Authorization (EUA) by the US Food and Drug Administration because of the need for widespread use in testing of samples from returning US travelers, and later in the outbreak, for use in the diagnosis of autochthonous cases.

Before 2016, commercial serologic test kits for ZIKV were absent. In the latter part of that year, three commercial ZIKV IgM ELISAs became available, one of which was emergency use authorized. The purpose of this study was to evaluate these kits in comparison to the reference diagnostic results, using a combination of ELISA and 90% plaque-reduction neutralization test (PRNT90) results. We aimed to determine sensitivity, specificity, and consistency of results across three geographically separated labs, using well-characterized blinded sera.

## 2. Materials and methods

### 2.1. Laboratories

Three laboratories participated in the study: Arbovirus Diseases Branch – Diagnostic and Reference Laboratory (ADB-DRL), CDC, Fort Collins, CO; Microbial Pathogenesis and Immune Response (MPIR) Laboratory, CDC, Atlanta, GA; Public Health Agency of Canada (PHAC), National Microbiology Laboratory, Winnipeg, Canada. All three participating laboratories were previously shown to be proficient in the use of the CDC Zika MAC-ELISA through participation in the CDC Arbovirus Proficiency Program. The ADB-DRL and PHAC laboratories function as arbovirus reference laboratories for the US and Canada, respectively.

### 2.2. Reference assays

Reference data were generated at the ADB-DRL for the serum panels described in Section 2.4 through a combination of CDC Zika MAC-ELISA and CDC DENV MAC-ELISA results (Lindsey et al., 2018), and confirmed by PRNT90 (Beaty et al., 1995) according to the CDC diagnostic algorithm https://www.cdc.gov/zika/pdfs/denvchikvzikv-testing-algorithm.pdf. The West Nile virus (WNV, family *Flaviviridae*, genus *Flavivirus*) IgM-positive, YFV (family *Flaviviridae*, genus *Flavivirus*) IgM-positive and chikungunya virus (CHIKV, family *Togaviridae*, genus *Alphavirus*) IgM-positive samples were tested by CDC MAC-ELISA and confirmed by PRNT90 for the respective viruses, and Zika and DENV MAC-ELISA results were also generated for these samples.

Inactivated whole ZIKV antigen generated in Vero E6 cells was used in the EUA Zika MAC-ELISA, and a combination of recombinant antigens of DENV serotypes 1–4 (E/prM proteins) made in COS-1 cells (Russell et al., 2007) was used in the DENV MAC-ELISA. Flavivirus group-reactive monoclonal antibody 6B6C-1-horseradish peroxidase (Tsai et al., 1987), custom-conjugated for the CDC by Jackson Immunoresearch (West Grove, PA), was used to detect reactions in both the Zika and DENV MAC-ELISAs.

The arbovirus MAC-ELISAs are qualitative tests, and the P/N ratio (optical density (OD) of the sample reacted on viral antigen/OD of negative control reacted on viral antigen) is not intended to compare results across samples. A P/N value of ≥3.0 is considered presumptive positive IgM; < 2.0 is considered negative, and results in between these values are considered equivocal. Positive, equivocal, and uninterpretable CDC Zika MAC-ELISA results were confirmed by PRNT90 using strain ZIKV strain PRVABC59, DENV serotype 1 (ChimeriVax YF/DEN chimera), and/or DENV serotype 2 (ChimeriVax YF/DEN chimera) (Guirakhoo et al., 2000). In the PRNT90, a sample was considered negative for neutralizing antibody to the challenge virus when 90% plaque-reduction was not observed at the lowest serum dilution used (1:10).

### 2.3. Kits

Kits from three manufacturers were used for the comparison. 1) ZIKV Detect™ IgM Capture ELISA (InBios International Inc., Seattle, WA) uses a recombinant ZIKV antigen made to the E/prM proteins, and also a cross-reactivity control antigen (CCA) made using recombinant DENV (Russell et al., 2007) and WNV prM/E proteins (Davis et al., 2001). This assay gained EUA in September, 2016. 2) NovaLisa® Zika IgM μ-capture ELISA (NovaTec Immundiagnostica GmbH, Dietzenbach, Germany) uses ZIKV nonstructural protein 1 (NS1) antigen. 3) The combined assays Anti-Zika Virus ELISA (IgM) and Anti-Zika Virus ELISA (IgG) (Euroimmun Medizinische Labordiagnostika AG (Lübeck, Germany), both use ZIKV NS1 antigen. For brevity in this paper, the kits are generally referred to as follows: InBios ZIKV Detect™ Capture IgM ELISA “InBios kit”; NovaTec NovaLisa® Zika IgM μ-capture ELISA “NovaTec kit”; Euroimmun Anti-Zika Virus (IgM) “Euroimmun IgM kit”; Euroimmun Anti-Zika Virus (IgG) “Euroimmun IgG kit”; and when used together, the Euroimmun IgM and IgG kits are referred to as “Euroimmun IgM + IgG kits”. The respective manufacturers generously provided all kits for this study.

Kit result interpretations varied according to manufacturer. Briefly, the InBios kit had four outcome categories: presumptive Zika positive, possible Zika positive, presumptive other flavivirus positive, and negative. The NovaTec kit had three outcome categories: positive, equivocal and negative. Euroimmun IgM and IgG kits each had three outcome categories: positive, borderline, and negative.

Kits were received at all three laboratories directly from the manufacturers in good condition, and each participating lab received the same lot number of kits from the individual manufacturers, with the exception of those from InBios, where it was necessary to obtain additional kits at a later date due to QC failures on using the first lot. Kits were stored at 4 °C prior to use, and used within a month of receipt and were well within the expiry dates. Results were classified according to the individual kit instructions and shared with the manufacturers. The Euroimmun IgM and IgG kit results were combined per the recommendation of Euroimmun, whereby a sample that gave positive results in either or both of the assays was classified as positive. Repeat testing of samples with equivocal or borderline results was not performed. Additional kits of an alternate lot number were obtained from NovaTec for purposes of testing Panels 2 and 3 (see Sections 2.4.2 and 2.4.3), and testing was performed at the ADB-DRL only for these panels.

All samples from Panel 1 (see Section 2.4.1) were tested using each kit at each of the three laboratories by adhering to the manufacturer’s instructions. Where options existed in the manufacturer’s instructions, all three labs agreed upon which option to use. For the NovaTec kit, the results acquisition option of using a reference wavelength of 630 nm was chosen. For the Euroimmun IgG kit, the quantitative results calculation option was used. Samples were run singly with all test kits. Plate validity parameters passed quality control prior to including results in the study, and plates were repeated as necessary to obtain valid results. Kits were subjectively assessed for ease-of-use, and the comparative features of each kit are presented in Table 1.

### 2.4. Serum samples

Panels were prepared in accordance with CDC Institutional Review Board protocol #6773 “Use of human specimens for laboratory research on arboviruses”.

#### 2.4.1. Panel 1

Panel 1 consisted of 289 blinded serum samples from symptomatic patients (pregnant and non-pregnant, all ages, male and female). The panel was designed to incorporate IgM-positive samples to the following viruses: ZIKV (N = 64); DENV diagnosed prior to the 2016 ZIKV epidemic (N = 47); flavivirus-positive samples obtained during the 2016 ZIKV epidemic where the infecting flavivirus was not identified (N = 62); CHIKV (N =10); YFV (N =10; WNV (N = 10). The panel also contained samples negative by MAC-ELISA to ZIKV, DENV, and to any other arboviruses tested during the diagnostic process (N = 78), along with samples that were uninterpretable in any of the MAC-ELISAs based on nonspecific reactivity (N = 8) identified using the normal antigen control portion of the test (Prince and Yeh, 2013). Samples from non-arboviral diseases were not included in the panel. Patients were primarily from the US, where the majority of ZIKV IgM-positive patients had recent travel histories to countries in which active ZIKV transmission was occurring. Dengue virus IgM-positive patients were from travelers to areas with active DENV transmission, and negative samples were from patients with varying backgrounds of domestic and international travel. Data were not available regarding country of origin of the patients or to where they may have previously resided or frequently traveled.

For the 64 confirmed ZIKV-positive samples, a range of P/N values (see Section 2.2 for P/N value definition) were included to discriminate the sensitivity of the commercial tests, and to represent the range of CDC Zika MAC-ELISA results seen during the course of the outbreak in 2016. Three had equivocal P/N values; six were in the range of 3.0 ≥ P/N < 5.0; eight were in the range of 5.0 ≥ P/N < 20, 13 were in the range of 20 ≥ P/N < 30; 34 were P/N ≥ 30. Samples used had been collected from 1 to 164 days post-symptom onset (DPO). Twenty-eight of these samples were taken < 7 DPO, and all but 4 (where sample volume was low) were tested by RT-PCR according to the CDC guidelines, in addition to the serologic methods. Of these 24 samples, 21 yielded negative results and 3 were equivocal. Paired acute and convalescent samples were not included in this analysis, due to the relative scarcity of paired samples received at the ADB-DRL. Details of Panel 1 samples and their reference results are presented in Supplemental Table 1. Sera were not heat-treated or processed in any way prior to use. Each sample was aliquoted into three tubes, frozen at −20 °C, and shipped overnight to participating laboratories. Samples were defrosted prior to use and maintained at 4 °C for the duration of the study.

#### 2.4.2. Panel 2

Following results analysis from the 3-lab study using Panel 1, a second blinded serum panel was compiled to look more critically at the range of dates after onset of symptoms that are appropriate for sample acquisition for use with the NovaTec and InBios kits. The Euroimmun kits were not included in this part of the study due to limited sample volume availability. This panel was also used to assess the usefulness of the NovaTec and InBios kits with serum samples from probable primary and secondary infections. Panel 2 consisted of 100 samples of mostly secondary flavivirus infections originating in Puerto Rico and obtained > 8 days post-onset of symptoms (50 ZIKV-positives and 50 DENV-positives, all confirmed by RT-PCR in earlier blood draws). These samples were tested at ADB-DRL only. Complete reference data (CDC Zika MAC-ELISA, CDC DENV MAC-ELISA and PRNT90) were generated for this second panel to differentiate probable primary from probable secondary ZIKV or DENV infections.

#### 2.4.3. Panel 3

Results from Panels 1 and 2 indicated that the NovaTec kit was highly sensitive between days 12 and 88 post-onset of symptoms. To investigate this more thoroughly, a third panel containing 30 primary Zika-positive samples taken days 12–88 post-onset of symptoms (PCR results not available), was tested using the NovaTec kit at the ADB-DRL only.

### 2.5. Statistical packages

The coefficient of variation (CV) and 95% confidence intervals (95% CI) were calculated using the unbiased estimates and the noncentral *t*-distribution with the “MBESS” package in R software (R Core Team, 2017). The Fleiss kappa statistic, used as a measure of inter-laboratory agreement, was calculated using the “raters” package in R software (R Core Team, 2017). Logistic regression using base R software was used to estimate the average sensitivities and specificities (and likelihood ratio 95% CI’s) for each test across labs. The need to incorporate a random lab effect was evaluated using the Akaiki Information Criterion (AIC) statistic and fitting with the “glmer” function in the “lme4” package in R.

## 3. Results

Raw data for Panel 1 and reference results are given in Supplemental Table 1.

### 3.1. Control measures

The Coefficient of variation (CV) of the positive and negative control ODs for each kit were compared for each laboratory, and results are shown in Table 2. The Euroimmun IgM kit generated the lowest mean CV for both the positive and negative controls (7.2 and 17.7 respectively), although CV’s varied notably among the three laboratories. The InBios kit had the mean highest CV for the positive control (28.0), and the NovaTec the highest CV for the negative control (30.5). The InBios kit also had a high CV for the negative control. Coefficients of variation were generally lower across the kits for the positive control.

### 3.2. Inter-laboratory agreement

Consistencies of Panel 1 results for the three ZIKV IgM kits across the three laboratories were analyzed. The results obtained by the NovaTec kit were the most consistent, where 95.2% of results agreed across all three laboratories. The Euroimmun IgM + IgG results agreed for 93.8% of the samples. The InBios kit exhibited significant variation where results agreed for 68.5% of the samples. Of the 91 samples that gave inconsistent results (defined as two-lab or zero-lab agreement) with the InBios kit, 34 samples were reference-result negative, 17 were reference-result DENV positive, 10 were reference-result ZIKV positive, and 30 were flavivirus or other arbovirus-positive. Results are shown in Table 3, including the Fleiss kappa statistic for each kit. The kappa statistic was very high (0.91) for both the NovaTec and Euroimmun IgM + IgG results, but was lower for the InBios kit (0.62).

### 3.3. Sensitivity and specificity

Sensitivity and specificity of the kits using Panel 1 was calculated and detailed results from the three participating laboratories, including 95% CI, are presented in Table 4. Briefly, when compared to the reference diagnostic results, the E protein-based InBios kit showed a mean sensitivity across the labs of 82.8% (95% CI 77.0, 87.7) when presumptive and possible Zika positive results were treated as positive. The NS1-based NovaTec kit had an average sensitivity of 69.8% (95% CI 63.1, 76.0). Results from the Euroimmun Zika IgM and IgG NS1-based kits were combined, per the manufacturer’s instructions, and had an average sensitivity of 62.5% (95% CI 55.5, 69.1). The sensitivities of the Euroimmun IgM and IgG kits alone were 53.1% (95% CI 46.1, 60.1) and 34.4% (95% CI 27.9, 41.3), respectively. In addition, sensitivity was analyzed using the reference values generated using only the CDC Zika MAC-ELISA, as shown in the Supplemental Table 2. These data were chiefly relevant for the InBios kit which uses E protein as the antigen source as does the CDC Zika MAC-ELISA, and here the sensitivity rose slightly to an average of 85.1%.

Specificity for each kit was calculated using data from the reference-negative samples. The mean specificities of the kits across the 3 labs were: InBios kit 83.3% (95% CI 78.0, 87.6), NovaTec kit 97.9% (95% CI 95.5, 99.2), and Euroimmun IgM + IgG 97.0% (95% CI 94.3, 98.7). Specificity was also calculated using 155 total samples comprising reference negatives plus samples from confirmed WNV, YFV, CHIKV and DENV infections. The average specificities were 72.7% (95% CI 68.5, 76.6), 97.6% (95% CI 96.0, 98.8), and 94.0% (95% CI 91.6, 95.9) for InBios, NovaTec and Euroimmun IgM + IgG kits, respectively. The Euroimmun IgM kit alone had a mean specificity of 97.4% (95% CI 95.7, 98.6), and the IgG kit had a mean specificity of 94.2% (95% CI 91.8, 96.1).

In estimating the average sensitivities or specificities for the tests across the three labs, the AIC was lower in each case for the models without a random lab effect, and further estimates for the random lab effects were either 0 or nearly so for all models. This indicated consistency among the labs in their diagnostic determinations. Estimates of the average values (and 95% CIs) computed using logistic regression are reported in the last column of Table 4.

### 3.4. Sensitivity and specificity of InBios and NovaTec kits with probable primary and secondary infections

Samples with data sufficient to classify them as probable ZIKV or DENV primary or secondary infections were used to determine the applicability of the InBios and NovaTec kits for use in DENV non-endemic and endemic regions. The infecting virus was identified for samples with positive RT-PCR results on acute paired samples from a DENV-endemic area, and probable primary or secondary status was determined for all samples by PRNT90. A monotypic response was indicative of a primary infection. A more extensive definition of probable versus confirmed primary and secondary ZIKV and DENV infections is discussed in Section 4. Specificity in this portion of the study was calculated using samples containing anti-DENV antibodies only, to simulate the performance of these kits in DENV-endemic areas. Table 5 compares the performance of the E protein-based InBios kit with the NS1 protein-based NovaTec kit on probable primary and secondary samples, where results are distinguished for RT-PCR-confirmed samples and PRNT90-only confirmed samples. Overall, the InBios kit had sensitivities of 85.9% and 100.0% for probable primary and secondary infections respectively. The NovaTec kit had sensitivities of 74.3% and 81.4% for probable primary and secondary infections respectively. For the InBios kit, specificity based upon DENV-positive samples were 38.7% and 9.3% for probable primary and secondary infections respectively. Specificity for the NovaTec kit was 100.0% for probable primary and 93.0% for probable secondary DENV infections.

### 3.5. Dates after onset of symptoms

**Fig. 1.**
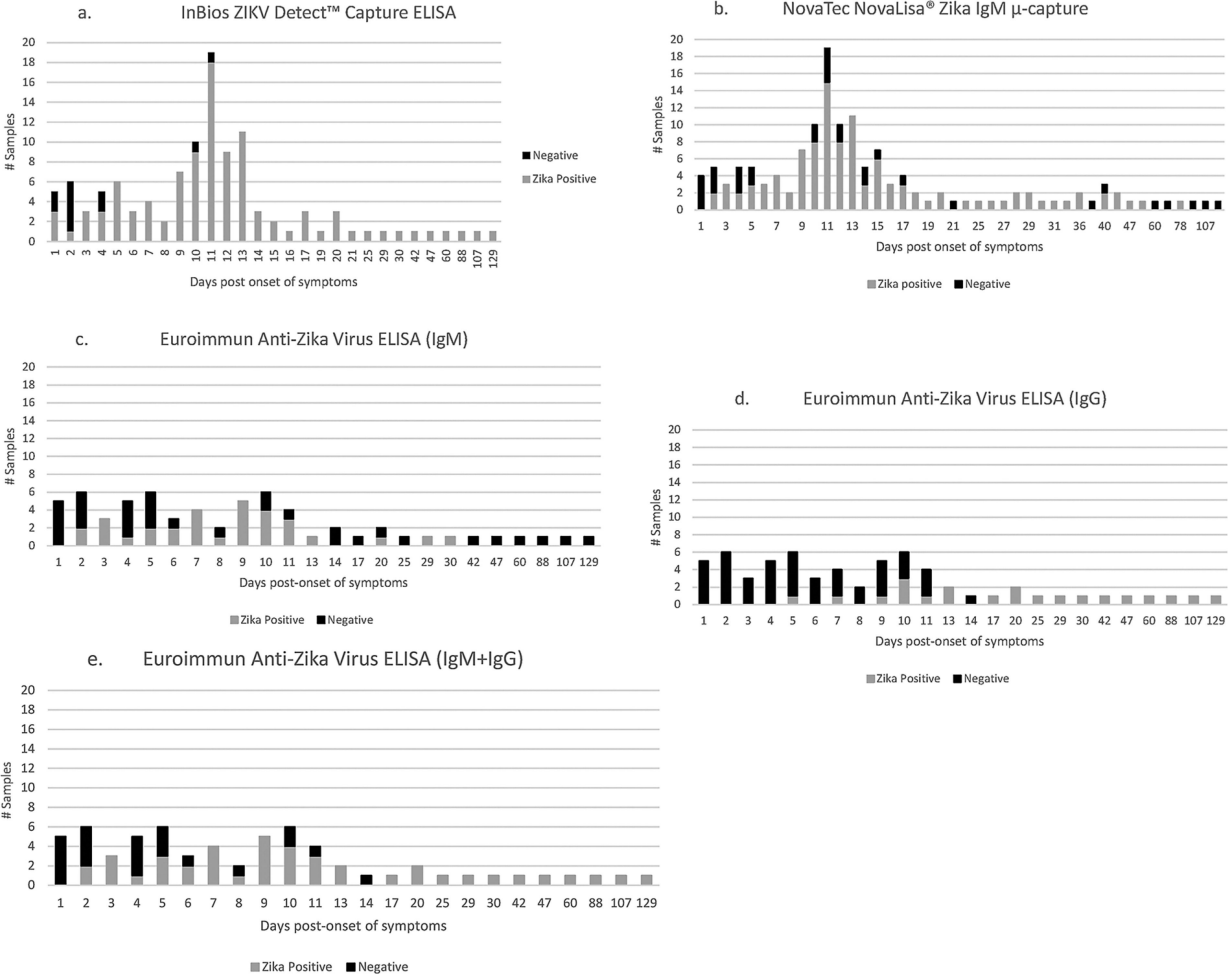
(a–e) Show the relationship between days post-onset of symptoms (DPO) and kit results for samples that tested ZIKV IgM-positive using reference methods. Numbers of samples tested at each time point are listed on the y-axis. Results in Fig. 1a for InBios ZIKV Detect™ IgM Capture ELISA were generated using serum Panels 1 and 2; results in Fig. 1b. for NovaTec NovaLisa® Zika Virus IgM μ-capture ELISA were generated using Panels 1, 2 and 3, where equivocals were classified as Zika Positive; results for Euroimmun Anti-Zika Virus ELISA IgM and IgG (Fig. 1c-e) were generated using Panel 1, where borderlines were classified as Zika Positive.

Results from Panels 1–3 from ABD-DRL only were examined to determine sensitivity by DPO for each of the kits, where only the confirmed ZIKV cases (N = 64) were included. Fig. 1a–e illustrates the number of ZIKV reference IgM-positive samples tested by day after onset of symptoms, and the number of associated positive and negative kit results. In Fig. 1a, ZIKV IgM-positive results for the InBios kit consisted of both presumptive Zika positive and possible Zika positive outcomes. The majority of the possible false negatives were observed in the first 4 days after onset. Fig. 1b shows that for the NovaTec kit, possible false negatives were seen in the first 5 days (41.1%) with proportionally less possible false negatives from day 6 until after day 78 (14.8%). Initially, results from Panel 1 indicated that from days 12–88 post-onset of symptoms, the sensitivity was 100.0% for the NovaTec kit (data not shown). However, the addition of Panels 2 and 3 revealed that some possible false negatives occurred in this date range. Results for the Euroimmun IgM and IgG kits are shown in Fig. 1c–e. The combined results of the IgM + IgG tests (Fig. 1e) showed false negativity over the first 14 days after onset of symptoms. The effect of combining the IgM and IgG results was to improve sensitivity following the first 14 days after onset, but no improvement was seen during the first 2 weeks after onset.

### 3.6. Uninterpretable results

The occasional uninterpretable result is to be expected in any test. Eight samples that were uninterpretable in the CDC Zika MAC-ELISA due to high background on the normal antigen control (three of which were PRNT90-positive to DENV serotypes 1 and 2 and none of which were ZIKV PRNT90 positive), were tested using each kit. For these 8 samples, the NovaTec kit gave 7 negative results in all labs, while the InBios kit gave 7 negative results in 2 labs, and a mixture of possible Zika positive, other flavivirus positive, and negative at the other lab. The Euroimmun IgM kit gave zero positive results in all labs, while the Euroimmun IgG kit gave negative results on 6 of the DENV-positive samples, where the 2 positive results were obtained on DENV-positive samples. The NovaTec and Euroimmun kits appeared to be of some use in sorting out the uninterpretable samples.

## 4. Discussion

The InBios ZIKV Detect™ IgM Capture ELISA, NovaTec NovaLisa® Zika Virus IgM μ-capture ELISA, and Euroimmun Anti-Zika Virus ELISAs (IgM and IgG) were compared to the CDC Zika MAC-ELISA and confirmatory PRNT90 results. The kit most comparable to the CDC Zika MAC-ELISA in test structure is the InBios kit, which uses an E/prM recombinant protein. While the reference results reported here for the CDC MAC-ELISA were obtained using inactivated whole virus antigen, the E-protein contains the immunodominant epitopes for ZIKV and therefore, these results were expected to be roughly comparable. In contrast, both the NovaTec kit and the Euroimmun IgM and IgG kits employ the NS1 protein, which would be expected to confer specificity to the ZIKV assays with respect to other flaviviruses, most importantly, DENV (Stettler et al., 2016). Thus, a limitation of this study is the comparison of assays that measure antibodies to non-identical parts of ZIKV for two of the three kits. Nevertheless, these kits are for use in the diagnosis of ZIKV, and therefore the performance comparison is useful to guide the contextual use of the assays using a larger number of samples than was available to the manufacturers at the time of assay development.

All the kits were easy to use, and all employed some colored reagents to act as a pipetting guide. All used 8-well strips to allow for multiple tests per kit and had adequate reagent volumes except for wash buffer when priming of the plate washer was required for multiple runs. The Euroimmun kits allowed the most samples to be tested and had the shortest times to completion. Analysis of results for the Euroimmun IgG kit was complex for the quantitative option due to the necessity of constructing a calibration curve for each plate, but simple for the semiquantitative option. The number of possible result interpretations was greatest for the InBios kit.

The NovaTec kit had the highest level of result agreement across the labs (95.2%), while the InBios kit had the least (68.5%). The variability for the InBios kit may be in part because of the incorporation of the CCA and the correspondingly more complex results algorithm. The InBios kit also exhibited some quality control (QC) failures in two of the laboratories. The failures were mostly due to the CCA to normal control antigen (NCA) ratio exceeding the QC limits, which were stringent.

Of the three kits, the InBios kit was the most sensitive compared to the CDC Zika MAC-ELISA; therefore, this kit is less likely than the other two to produce false negative results. However, for the InBios kit, sensitivity was poor for samples with low positive CDC Zika MAC-ELISA results, and in samples taken within 6 days after onset of symptoms. This may be largely due to the shortened incubation time of the ZIKV antigen in the InBios kit (1 h) as compared to the overnight antigen incubation in the CDC MAC-ELISA. Of note, when comparing the antigen incubation conditions of the InBios ZIKV Detect IgM Capture ELISA kit to either the InBios DENV Detect IgM Capture ELISA or the InBios JE Detect kit, a lack of sensitivity was not noticed with the DENV kit (Hunsperger et al., 2016), but was noticed with the JE kit (Robinson et al., 2010). The lower specificity of the InBios kit, and the high proportion of reference-negative samples yielding inconsistent results across the three labs, suggest a possibility of false positivity when using this kit as a diagnostic tool. In addition, the lack of a lower limit of positivity cut-off based upon results from a negative human serum control may have contributed to some false positivity. The CCA is included in this kit to differentiate ZIKV from non-ZIKV flaviviruses. Most of the false-positive results with the InBios kit were identified as “possible Zika positive”. The high number of DENV samples identified as possible ZIKV positive as opposed to presumptive other flavivirus suggests that the utility of the CCA was not being leveraged as well as it might. A change in the results interpretation algorithm for this kit may improve the specificity. InBios International received approval in May 2018 from the FDA for EUA of ZIKV Detect 2.0 IgM ELISA, which incorporates a new algorithm and some methodological changes.

The NovaTec kit and the Euroimmun IgM + IgG kits had lower sensitivity but higher specificity than the InBios kit. The reason for these lower sensitivities is unclear; it is possible that relatively low amounts of anti-NS1 antibody are produced in some patients, but this possibility has not been verified. The combined use of the Euroimmun IgM and IgG kits improved sensitivity over the individual tests with only minor deleterious effect on specificity when the DENV samples in Panel 1 were included in the denominator. Although the dengue IgM-positive samples used in Panel 1 were obtained prior to the 2016 ZIKV epidemic, it cannot be excluded that some dengue patients had contracted ZIKV infection when traveling to DENV- (and ZIKV-) endemic areas. Neutralization test results were unavailable for these samples; therefore, there is a possibility that these patients had prior infection or co-infection with ZIKV. This is a limitation of the study. The Euroimmun IgM kit used alone suffered a surprising lack of sensitivity, which may be due to the test configuration where a recombinant ZIKV NS1 antigen is coated directly onto the wells as opposed to being captured. The collective experience of ADB-DRL has shown this method to be sub-optimal. The combination IgM + IgG approach recommended by Euroimmun may be of use where ZIKV is new to a particular community, but once the virus becomes endemic, the combined result will not provide information regarding whether the result reflects recent or past ZIKV infections. This could also be a problem for travelers that frequent flavivirus endemic areas. However, reliable IgM and IgG results could be useful when immune status is needed for planned pregnancy or for vaccine studies. IgM is sometimes diminished or absent in secondary flavivirus infections, where a boost in IgG to the primary infecting virus is seen (Sa Ngasang et al., 2006). The combination of IgM and IgG tests may serve to mitigate false negative results in dengue endemic areas, though further testing would be required (Tsai et al., 2015).

The established method for distinguishing primary from secondary DENV infections requires the use of paired samples (de Souza et al., 2007; Innis et al., 1989) and IgM/IgG ratios, which were not possible to obtain for the majority of samples in this analysis. However, the comparison of neutralization titers, where samples from primary infections exhibit monotypic responses, and secondary infections exhibit high titers to multiple DENV serotypes (and often to multiple flaviviruses) is widely considered an indication of infection status (Tsai et al., 2015; Vorndam and Beltran, 2002). A standard has not been established to define primary Zika infections, but a monotypic neutralization response can reasonably be expected to reflect primary infections, especially if the patient is not from a DENV-endemic area. Interestingly, the sensitivities of both the InBios and NovaTec kits were slightly greater overall for probable secondary ZIKV infections than for primary. This is counterintuitive as IgM to the immunodominant E protein is sometimes diminished in secondary infections, although the kinetics of the IgM response to the NS1 antigen is unknown. The specificity of the InBios kit based on DENV infections only was low overall (probable primary infections 38.7%; secondary infections 9.3%). This was not surprising due to the known reactivity of antibodies generated to flavivirus group-reactive epitopes on the E protein, especially in secondary infections (Tsai et al., 2015). Specificity was reduced slightly in the probable secondary infections tested with the NovaTec kit. The lack of comparison of the Euroimmun tests for use with primary and secondary infections is a limitation of the study, as is the lack of samples from primary and secondary infections identified using IgM/IgG ratios on paired sera.

The three-lab kit comparison included samples from undifferentiated flavivirus infections (see Supplemental Table 1). These samples formed part of the across-laboratory agreement data (Supplemental Table 2), and were included in Panel 1 because a diagnosis of “flavivirus” is a common occurrence using the current tests where positive RT-PCR results are absent for a patient. The sensitivity and specificity data generated for the NS1 protein-based kits used in this study provide an indication of how a portion of samples with the current diagnosis of flavivirus could be assigned a more informative result. The sensitivity of the E protein-based InBios kit identifies a sample as potentially being ZIKV IgM-positive, but the relative lack of specificity of the E protein-based test would preclude further inferences as to the infecting virus. The inclusion of an NS1 result for samples with a diagnosis of “flavivirus” using the kits investigated here would indicate that patients with NS1-positive results have likely been exposed to ZIKV.

All three assays evaluated here lacked sensitivity in the first 5 days after onset of symptoms, and therefore are more suitable for use beyond this point. Acute samples are appropriate for testing by RT-PCR. Beyond approximately day 40 after onset of symptoms, the NovaTec and Euroimmun IgM kits produced generally negative results, indicating a lack of sensitivity in this range. The possibility exists that anti-NS1 IgM antibodies do not persist in the serum for as long as anti-E protein antibodies have been shown to persist (Paz-Bailey et al., 2017). The number of samples beyond day 40 was low for this study and is a limitation.

Immunoglobulin M antibodies elicited to the E proteins of DENV and other flaviviruses are highly cross-reactive with ZIKV E protein. This indicates that E protein-based Zika IgM assays are only appropriate for use where positive results are informative. Samples from patients living in DENV non-endemic areas, and newborns from DEN-endemic areas, are suitable candidates for E-protein-based testing. These data suggest that the NS1-based assays evaluated here are unsuitable as standalone tests due to the possibility of false-negative results even after the first week after onset of symptoms. However, due to their excellent specificity, they could potentially replace the highly complex PRNT90 for ZIKV infection confirmation in some cases, where a positive result is confirmatory but a negative result would be inconclusive. Due to the ease of use of the kits, this could be of great advantage, especially in labs where the PRNT90 is not part of the diagnostic repertoire, and would reduce turnaround time. For the NS1-based assays described here to be used clinically in the United States, FDA approval or Emergency-Use Authorization would need to be acquired.

The PRNT90 is unsuitable for use in countries with endemic DENV where the majority of ZIKV infections are likely to be secondary, making PRNT90 uninformative due to the cross-reactive nature of the flavivirus neutralizing antibodies. The use of the NS1 IgM ELISAs investigated here in these regions could allow ZIKV infections to be identified in a portion of the cases. Currently, identification of ZIKV infection relies on RT-PCR, which is only useful in the acute stage (CDC). To avoid unnecessary cost in the use of NS1 IgM ELISAs, serum would ideally need to be drawn between day 6 and 40 post-onset of symptoms.

The findings of this study are consistent with recently published data (Safronetz et al., 2017; L’Huillier et al., 2017; Lustig et al., 2017; Granger et al., 2017; Kadkhoda et al., 2017), and expand on those results. The potential use of the assays investigated here may not extend to other E protein-based or NS1-based assay formats or kits for ZIKV Any testing algorithm should be based on rigorously and independently verified performance characteristics of the assays involved. The acquisition of appropriately timed, clinically relevant and/or travel-related samples is critical to avoid the over-occurrence of false positive results in any assay. Further work is needed using larger numbers of well-defined samples to address the limitations of this study and truly elucidate the use of each of these tests, and in particular to investigate the performance of ZIKV NS1 protein-based assays. These results will be of use to domestic and international diagnostic laboratories as they transition to the use of commercial tests, and to add to the literature that informs public health decisions related to ZIKV.

## Supplementary Material

1

2

## Figures and Tables

**Table 1:** Comparison of kit features.

Kit feature	InBios	NovaTec	Euroimmun IgM	Euroimmun IgG
Specimen type	Serum	Serum, plasma	Serum, plasma	Serum, plasma
Number of samples/kit (in single replicates)	28	89	93	Semiquantitative – 93 Quantitative – 91
Sample volume	4 μl	10 μl	10 μl	10 μl
Sample dilution	1:100	1:100	1:101	1:101
Number of wells	96 (8-well strips)	96 (8-well strips)	96 (8-well strips)	96 (8-well strips)
Duration of test (for 1 full plate)	∼4–5h	∼3–4 h	∼2.5–3h	∼2.5–3 h
Detects immunoglobulin antibody	IgM	IgM	IgM	IgG
Antigen type(s)	ZIKV E/prM; flavivirus cross-reactive; normal	ZIKV NS1 (HRP-conjugated)	ZIKV NS1	ZIKV NS1

Abbreviations: ZIKV–Zika virus; E–envelope; prM–pre-membrane; HRP–horseradish peroxidase; NS1–non-structural protein 1.

**Table 2:** Coefficient of variation (95% CIs) for positive and negative controls based on Zika antigen optical density values.

ELISA kit	Lab					
	ADB-DRL		MPIR		PHAC	
	Positive	Negative	Positive	Negative	Positive	Negative
InBios	47.13 (30.65, 95.42)	25.74 (17.31, 46.45)	18.43 (12.69, 31.40)	20.77 (14.27, 35.61)	18.52 (12.55, 32.63)	30.43 (20.34, 56.01)
NovaTec	10.02 (5.33, 36.18)	27.84 (14.55, 123.40)	15.62 (8.28, 58.74)	33.66 (17.44, 168.45)	21.70 (11.43, 87.35)	29.86 (15.57, 137.58)
Euroimmun IgM	4.58 (2.51, 16.15)	5.45 (2.98, 19.27)	7.06 (3.87, 25.13)	13.71 (7.27, 50.71)	9.92 (5.32, 35.77)	34.06 (17.64, 172.12)
Euroimmun IgG	9.53 (5.21, 34.30)	7.82 (4.28, 27.92)	13.70 (7.27, 50.68)	8.19 (4.48, 29.27)	8.60 (4.71, 30.81)	70.40 (33.58,∞)

Abbreviations: ADB-DRL–Arbovirus Diseases Branch-Diagnostic and Reference Laboratory; MPIR–Microbial Pathogenesis and Immune Response Laboratory; PHAC–Public Health Agency of Canada.

**Table 3:** Agreement between laboratories for each kit using Panel 1.

Kit	Lab Agreement N (%)			Fleiss’ kappa statistic (95% CI)
	All three agreed	Two agreed	No agreement	
InBios	198 (68.5)	86 (29.8)	5 (1.7)	0.62 (0.57, 0.68)
NovaTec	275 (95.2)	11 (3.8)	3 (1.0)	0.91 (0.85, 0.97)
Euroimmun IgM	260 (90.0)	23 (8.0)	6 (2.1)	0.78 (0.72, 0.84)
Euroimmun IgG	270 (93.4)	16 (5.5)	3 (1.0)	0.89 (0.83, 0.95)
Euroimmun IgM & IgG	271 (93.8)	18 (6.2)	0 (0.0)	0.91 (0.85, 0.98)

**Table 4:** Sensitivity and specificity of kits tested at 3 laboratories compared to confirmed reference results.

Overall CDC Diagnosis			Laboratory results												
			ADB-DRL				PHAC				MPIR				Combined
				
Test	Measure	N	POS	EQ	NEG	%	POS	EQ	NEG	%	POS	EQ	NEG	%	% (95% CI)
InBios^a^	Sensitivity	64	54^b^	NA	9	84.4	52^b^	NA	11	81.3	53^b^	NA	11	82.8	82.8 (77.0, 87.7)
	Specificity^c^	78	14^b^	NA	57	73.1	6^b^	NA	71	91.0	10^b^	NA	67	87.2	83.3 (78.0, 87.6)
	Specificity^d^	155	45^b^	NA	110^e^	71.0	40^b^	NA	115^d^	74.2	42^b^	NA	113^e^	72.9	72.7 (68.5,76.6)
NovaTec	Sensitivity^f^	64	40	3	21	67.2	40	5	19	70.3	45	1	18	71.9	69.8 (63.1, 76.0)
	Specificity^c^	78	2	0	76	97.4	1	0	77	98.7	2	0	76	97.4	97.9 (95.5, 99.2)
	Specificity^d^	155	3	0	152	98.1	2	0	153	98.7	6	0	149	96.1	97.6 (96.0, 98.8)
Euroimmun IgM	Sensitivity^e^	64	28	4	32	50.0	30	3	31	51.6	33	4	27	57.8	53.1 (46.1, 60.1)
	Specificity^c^	78	0	1	77	98.7	1	1	76	97.4	3	1	74	96.2	97.0 (94.3, 98.7)
	Specificity^d^	155	2	1	152	98.1	2	1	152	98.1	5	1	149	96.8	97.4 (95.7, 98.6)
Euroimmun IgG	Sensitivity^f^	64	20	1	43	32.8	20	0	44	31.3	22	3	39	39.1	34.4 (27.9, 41.3)
	Specificity^c^	78	1	0	77	98.7	0	1	77	98.7	2	0	76	97.4	98.3 (96.1, 99.5)
	Specificity^d^	155	5	2	148	95.5	5	3	147	94.8	9	3	143	94.2	94.2 (91.8, 96.1)
Euroimmun IgM + IgG	Sensitivity^f^	64	39	NA	25	60.9	39	NA	25	60.9	42	NA	22	65.6	62.5 (55.5, 69.1)
	Specificity^c^	78	1	NA	77	98.7	1	NA	77	98.7	5	NA	73	93.6	97.0 (94.3, 98.7)
	Specificity^d^	155	7	NA	148	95.5	7	NA	148	95.5	14	NA	141	91.0	94.0 (91.6, 95.9)

a For InBios, positive plus negative numbers do not add up to N in some cases, because of the “Presumptive Other Flavivirus” category.

b For the InBios kit, positive results are considered both “Presumptive Zika positive” and “Possible Zika positive”.

c Specificity denominator consists of true negative samples only.

d Specificity denominator consists of 10 YF, 10 WN, 10 CHIK, 47 DEN and 78 NEG samples.

e Negative plus Presumptive Other Flavivirus results are classified as a negative result.

f Sensitivity was calculated where equivocal results were classified as positive.

**Table 5:** Sensitivity and specificity of InBios and NovaTec kits using samples from probable primary and secondary infections.

A. Sensitivity using confirmed ZIKV positive samples		InBios				NovaTec				
Probable status	Tests used	N^a^	POS^b^	NEG	% Sensitivity (95% CI)	N^c^	POS	EQ	NEG	% Sensitivity (95% CI)

Primary	RT-PCR, PRNT90	7	7	0	100.0 (64.6, 100)	7	7	0	0	100.0 (64.6, 100.0)
Primary	PRNT 90	64	54	10	84.4 (73.6, 91.3)	94	64	4	26	72.3 (62.6, 80.4)
Overall^d^ 1° sensitivity		71	61	10	85.9 (76.0, 92.2)	101	71	4	26	74.3 (65.0, 81.8)
Secondary	RT-PCR, PRNT90	43	43	0	100.0 (91.8, 100.0)	43	34	1	8	81.4 (67.4, 90.3)
Secondary	PRNT90	0	0	0	No data	0	0	0	0	No data
Overall^d^ 2° sensitivity		43	43	0	100.0 (91.8, 100.0)	43	34	1	8	81.4 (67.4, 90.3)
Overall sensitivity		114	104	10	91.2 (84.6, 95.2)	144	105	5	34	76.4 (68.8, 82.6)

B. Specificity using confirmed DENV positive samples		InBios				NovaTec				
DEN	Tests used	N^a^	POS^b^	NEG	% Specificity (95% CI)	N^c^	POS	EQ	NEG	% Specificity (95% CI)

Primary	RT-PCR, PRNT90	13	10	3	23.1 (8.2, 50.3)	13	0	0	13	100.0 (77.2, 100.0)
Primary	PRNT90	18	9	9	50.0 (29.0, 71.0)	18	0	0	18	100.0 (82.4, 100.0)
Overall^d^ 1° specificity		31	19	12	38.7 (23.7, 56.2)	31	0	0	31	100.0 (89.0, 100.0)
Secondary	RT-PCR, PRNT90	37	35	2	5.4 (1.5, 17.7)	37	2	1	34	91.9 (78.7, 97.2)
Secondary	PRNT90	6	4	2	33.3 (9.7, 70.0)	6	0	0	6	100.0 (61.0, 100.0)
Overall^d^ 2° specificity		43	39	4	9.3 (3.7, 21.6)	43	2	1	40	93.0 (81.4, 97.6)
Overall specificity		74	58	16	21.6 (13.8, 32.3)	74	2	1	71	95.9 (88.7, 98.6)

a Using results from Panels 1 and 2.

b Presumptive and possible Zika positives classified as POS.

c Using results from Panels 1, 2 and 3.

d Refers to probable infection status.

## References

[R1] Beaty BJ, Claisher CH, Shope RE, Arboviruses, 1995. In: Lennette EH, Lennette DA (Eds.), Diagnostic Procedures for Viral, Rickettsial and Chlamydial Infections, 7th ed. American Public Health Association, Washington, DC, pp. 189–212.

[R2] Brasil P, Calvet G, Siqueira A, Wakimoto M, de Sequeira P, Nobre A, Quintana Md.S.B., Mendonça M.C.Ld., Lupi O, de Souza R, Romero C, Zogbi H, Bressan Cd.S., Alves S, Lourenço-de-Oliveira R, Nogueira RMR, Carvalho M, de Filippis AMB, Jaenisch T, 2016. Zika virus outbreak in Rio de Janeiro, Brazil: clinical characterization, epidemiological and virological aspects. PLoS Negl. Trop. Dis 10, e0004636. 27070912 10.1371/journal.pntd.0004636 PMC4829157

[R3] Cao Lormeau V-M, Roche C, Teissier A, Robin E, Berry A-L, Mallet H-P, Sall A, Musso D, 2014. Zika virus, French polynesia, South pacific, 2013. Emerg. Infect. Dis 20, 1085–1086. 24856001 10.3201/eid2006.140138 PMC4036769

[R4] Davis BS, Chang GJ, Cropp B, Roehrig JT, Martin DA, Mitchell CJ, Bowen R, Bunning ML, 2001. West Nile virus recombinant DNA vaccine protects mouse and horse from virus challenge and expresses in vitro a noninfectious recombinant antigen that can be used in enzyme-linked immunosorbent assays. J. Virol 75, 4040–4047. 11287553 10.1128/JVI.75.9.4040-4047.2001 PMC114149

[R5] de Souza VAUF, Tateno A, Oliveira R, Domingues R, Araújo E, Kuster G, Pannuti C, 2007. Sensitivity and specificity of three ELISA-based assays for discriminating primary from secondary acute dengue virus infection. J. Clin. Virol 39, 230–233. 17509934 10.1016/j.jcv.2007.04.005

[R6] Dick GWA, Kitchen SF, Haddow AJ, 1952. Zika Virus (I). Isolations and serological specificity. Trans. R. Soc. Trop. Med. Hyg 46, 509–520. 12995440 10.1016/0035-9203(52)90042-4

[R7] Duffy M, Chen T-H, Hancock WT, Powers A, Kool J, Lanciotti R, Pretrick M, Marfel M, Holzbauer S, Dubray C, Guillaumot L, Griggs A, Bel M, Lambert A, Laven J, Kosoy O, Panella A, Biggerstaff B, Fischer M, Hayes E, 2009. Zika virus outbreak on Yap Island, Federated States of Micronesia. N. Engl. J. Med 360, 2536–2543. 19516034 10.1056/NEJMoa0805715

[R8] Fitzgerald B, Boyle C, Honein M, 2018. Birth defects potentially related to Zika virus infection during pregnancy in the United States. JAMA: J. Am. Med. Assoc 319, 1195–1196. 10.1001/jama.2018.0126 PMC5988243 29372233

[R9] Granger D, Hilgart H, Misner L, Christensen J, Bistodeau S, Palm J, Strain AK, Konstantinovski M, Liu D, Tran A, Theel ES, 2017. Serologic testing for Zika virus: comparison of three Zika virus IgM-screening enzyme-linked immunosorbent assays and initial laboratory experiences. J. Clin. Microbiol 55, 2127–2136. 28446573 10.1128/JCM.00580-17 PMC5483914

[R10] Guirakhoo F, Weltzin R, Chambers TJ, Zhang ZX, Soike K, Ratterree M, Arroyo J, Georgakopoulos K, Catalan J, Monath TP, 2000. Recombinant chimeric yellow fever-dengue type 2 virus is immunogenic and protective in nonhuman primates. J. Virol 74, 5477–5485. 10823852 10.1128/jvi.74.12.5477-5485.2000 PMC112032

[R11] Gulland A, 2016. Zika virus is a global public health emergency, declares WHO. BMJ Br. Med. J 352, i657. 26839247 10.1136/bmj.i657

[R12] Hunsperger E, Muñoz Jordán J, Beltran M, Colón C, Carrión J, Vazquez J, Acosta L, Medina Izquierdo J, Horiuchi K, Biggerstaff B, Margolis H, 2016. Performance of dengue diagnostic tests in a single-specimen diagnostic algorithm. J. Infect. Dis 214, 836–844. 26984143 10.1093/infdis/jiw103

[R13] Innis BL, Nisalak A, Nimmannitya S, Kusalerdchariya S, Chongswasdi V, Suntayakorn S, Puttisri P, Hoke CH, 1989. An enzyme-linked immunosorbent assay to characterize dengue infections where dengue and Japanese encephalitis cocirculate. Am. J. Trop. Med. Hyg 40, 418–427. 2540664 10.4269/ajtmh.1989.40.418

[R14] Kadkhoda K, Gretchen A, Racano A, 2017. Evaluation of a commercially available Zika virus IgM ELISA: specificity in focus. Diagn. Microbiol. Infect. Dis 88, 233–235. 28478111 10.1016/j.diagmicrobio.2017.04.002

[R15] Khawar W, Bromberg R, Moor M, Lyubynska N, Mahmoudi H, 2017. Seven cases of Zika virus infection in South Florida. Curēus 9, e1099. 28413745 10.7759/cureus.1099 PMC5392035

[R16] Lanciotti R, Kosoy O, Laven J, Velez J, Lambert A, Johnson A, Stanfield S, Duffy M, 2008. Genetic and serologic properties of Zika virus associated with an epidemic, Yap State, Micronesia, 2007. Emerg. Infect. Dis 14, 1232–1239. 18680646 10.3201/eid1408.080287 PMC2600394

[R17] Lindsey N, Staples JE, Powell K, Rabe I, Fischer M, Powers A, Kosoy O, Mossel E, Munoz Jordan J, Beltran M, Hancock WT, Toews K-A, Ellis E, Ellis B, Panella A, Basile A, Calvert A, Laven J, Goodman C, Gould C, Martin S, Thomas J, Villanueva J, Mataia M, Sciulli R, Gose R, Whelen AC, Hills S, 2018. Ability to serologically confirm recent Zika virus infection in areas with varying past incidence of dengue virus infection in the United States and U.S. Territories in 2016. J. Clin. Microbiol 56. 10.1128/JCM.01115-17 PMC5744200 29093104

[R18] L’Huillier A, Hamid Allie A, Kristjanson E, Papageorgiou L, Hung S, Wong C, Stein D, Olsha R, Goneau L, Dimitrova K, Drebot M, Safronetz D, Gubbay J, 2017. Evaluation of euroimmun anti-Zika virus IgM and IgG enzyme-linked immunosorbent assays for Zika virus serologic testing. J. Clin. Microbiol 55, 2462–2471. 28566316 10.1128/JCM.00442-17 PMC5527425

[R19] Lustig Y, Zelena H, Venturi G, Van Esbroeck M, Rothe C, Perret C, Koren R, Katz-Likvornik S, Mendelson E, Schwartz E, 2017. Sensitivity and kinetics of an NS1-based Zika virus enzyme-linked immunosorbent assay in Zika virus-infected travelers from Israel, the Czech Republic, Italy, Belgium, Germany, and Chile. J. Clin. Microbiol 55, 1894–1901. 28381608 10.1128/JCM.00346-17 PMC5442546

[R20] Paz-Bailey G, Rosenberg ES, Doyle K, Munoz-Jordan J, Santiago G, Klein L, Perez-Padilla J, Medina FA, Waterman S, Garcia Gubern C, Alvarado LI, Sharp T, 2017. Persistence of Zika Virus in Body Fluids — Preliminary Report. 10.1056/NEJMoa1613108 PMC5831142 28195756

[R21] Prince H, Yeh C, 2013. Reactivity of human IgM binding murine monoclonal 6B6C1 (IgG2a) with other murine monoclonal IgG antibodies. J. Clin. Lab. Anal 27, 27–30. 23292827 10.1002/jcla.21557 PMC6807545

[R22] R Core Team, 2017. R: A language and environment for statistical computing. R Foundation for Statistical Computing, Vienna, Austria. URL https://www.R-project.org/.

[R23] Robinson J, Featherstone D, Vasanthapuram R, Biggerstaff B, Desai A, Ramamurty N, Chowdhury A, Sandhu H, Cavallaro K, Johnson B, 2010. Evaluation of three commercially available Japanese encephalitis virus IgM enzyme-linked immunosorbent assays. Am. J. Trop. Med. Hyg 83, 1146–1155. 21036854 10.4269/ajtmh.2010.10-0212 PMC2963986

[R24] Russell B, Velez J, Laven J, Johnson A, Chang G-J, Johnson B, 2007. A comparison of concentration methods applied to non-infectious flavivirus recombinant antigens for use in diagnostic serological assays. J. Virol. Methods 145, 62–70. 17570536 10.1016/j.jviromet.2007.05.008

[R25] Safronetz D, Sloan A, Stein D, Mendoza E, Barairo N, Ranadheera C, Scharikow L, Holloway K, Robinson A, Traykova Andonova M, Makowski K, Dimitrova K, Giles E, Hiebert J, Mogk R, Beddome S, Drebot M, 2017. Evaluation of 5 commercially available Zika virus immunoassays. Emerg. Infect. Dis 23, 1577–1580. 28665268 10.3201/eid2309.162043 PMC5572859

[R26] Sa Ngasang A, Anantapreecha S, A-Nuegoonpipat A, Chanama S, Wibulwattanakij S, Pattanakul K, Sawanpanyalert P, Kurane I, 2006. Specific IgM and IgG responses in primary and secondary dengue virus infections determined by enzyme-linked immunosorbent assay. Epidemiol. Infect 134, 820–825. 16371180 10.1017/S0950268805005753 PMC2870461

[R27] Stettler K, Beltramello M, Espinosa D, Graham V, Cassotta A, Bianchi S, Vanzetta F, Minola A, Jaconi S, Mele F, Foglierini M, Pedotti M, Simonelli L, Dowall S, Atkinson B, Percivalle E, Simmons C, Varani L, Blum J, Baldanti F, Cameroni E, Hewson R, Harris E, Lanzavecchia A, Sallusto F, Corti D, 2016. Specificity, cross-reactivity, and function of antibodies elicited by Zika virus infection. Science 353, 823–826. 27417494 10.1126/science.aaf8505

[R28] Tognarelli J, Ulloa S, Villagra E, Lagos J, Aguayo C, Fasce R, Parra B, Mora J, Becerra N, Lagos N, Vera L, Olivares B, Vilches M, Fernández J, 2016. A report on the outbreak of Zika virus on Easter Island, South Pacific, 2014. Arch. Virol 161, 665–668. 26611910 10.1007/s00705-015-2695-5

[R29] Tsai TF, Bolin RA, Montoya M, Bailey RE, Francy DB, Jozan M, Roehrig JT, 1987. Detection of St. Louis encephalitis virus antigen in mosquitoes by capture enzyme immunoassay. J. Clin. Microbiol 25, 370–376. 3029170 10.1128/jcm.25.2.370-376.1987 PMC265902

[R30] Tsai W-Y, Durbin A, Tsai J-J, Hsieh S-C, Whitehead S, Wang W-K, 2015. Complexity of neutralizing antibodies against multiple dengue virus serotypes after heterotypic immunization and secondary infection revealed by in-depth analysis of cross-reactive antibodies. J. Virol 89, 7348–7362. 25972550 10.1128/JVI.00273-15 PMC4473561

[R31] Vorndam V, Beltran M, 2002. Enzyme-linked immunosorbent assay-format microneutralization test for dengue viruses. Am. J. Trop. Med. Hyg 66, 208–212. 12135295 10.4269/ajtmh.2002.66.208

[R32] Wikan N, Smith D, 2016. Zika virus: history of a newly emerging arbovirus. Lancet Infect. Dis 16, e119–e126. 27282424 10.1016/S1473-3099(16)30010-X

[R33] Zanluca C, Melo V.C.Ad., Mosimann ALP, Santos CNDD, Luz K, 2015. First report of autochthonous transmission of Zika virus in Brazil. Memórias do Instituto Oswaldo Cruz 110, 569–572. 26061233 10.1590/0074-02760150192 PMC4501423

